# Laser Safety—What Is the Laser Hazard Distance for an Electro-Optical Imaging System?

**DOI:** 10.3390/s23167033

**Published:** 2023-08-08

**Authors:** Gunnar Ritt

**Affiliations:** Fraunhofer IOSB, Gutleuthausstr. 1, 76275 Ettlingen, Germany; gunnar.ritt@iosb.fraunhofer.de

**Keywords:** laser safety, sensor damage, sensor dazzle

## Abstract

Laser safety is an important topic. Everybody working with lasers has to follow the long-established occupational safety rules to prevent people from eye damage by accidental irradiation. These rules comprise, for example, the calculation of the Maximum Permissible Exposure (MPE), as well as the corresponding laser hazard distance, the so-called Nominal Ocular Hazard Distance (NOHD). At exposure levels below the MPE, laser eye dazzling may occur and is described by a quite new concept, leading to definitions such as the Maximum Dazzle Exposure (MDE) and to its corresponding Nominal Ocular Dazzle Distance (NODD). In earlier work, we defined exposure limits for sensors corresponding to those for the human eye: The Maximum Permissible Exposure for a Sensor, ${MPE}_{S}$, and the Maximum Dazzle Exposure for a Sensor, ${MDE}_{S}$. In this publication, we report on our continuative work concerning the laser hazard distances arising from these exposure limits. In contrast to the human eye, unexpected results occur for electro-optical imaging systems: For laser irradiances exceeding the exposure limit, ${MPE}_{S}$, it can happen that the laser hazard zone does not extend directly from the laser source, but only from a specific distance to it. This means that some scenarios are possible where an electro-optical imaging sensor may be in danger of getting damaged within a certain distance to the laser source but is safe from damage when located close to the laser source. This is in contrast to laser eye safety, where it is assumed that the laser hazard zone always extends directly from the laser source. Furthermore, we provide closed-form equations in order to estimate laser hazard distances related to the damaging and dazzling of the electro-optical imaging systems.

## 1. Introduction

When an electro-optical imaging system (e.g., a surveillance camera) is illuminated with laser radiation of appropriate wavelength and power/pulse energy, the imaging system may become incapacitated [1,2,3]. Incapacitation means that the imaging system can no longer fulfill its intended task, either due to reversible (dazzle) or irreversible (damage) effects. The result of dazzling and/or damaging of imaging systems by laser radiation can be irritating to an observer but may be of less importance in the civilian sector, unlike in military operations, where laser attacks pose a severe threat [4,5,6,7,8,9] and may disrupt operations. In order to inform people about laser hazards, it would be beneficial to have the ability to perform laser safety calculations for imaging systems that ideally follow the existing concepts of laser eye safety.

The long-established occupational safety rules to prevent people from eye damage by accidental irradiation comprise the calculation of the Maximum Permissible Exposure (MPE) and its corresponding hazard distance, the Nominal Ocular Hazard Distance (NOHD) [10,11], in order to choose the proper laser safety eyewear [12,13]. Corresponding quantities to MPE and NOHD but related to the reversible effect of laser dazzle of the human eye are the Maximum Dazzle Exposure (MDE) and Nominal Ocular Dazzle Distance (NODD) [14,15,16].

**Figure 1:**
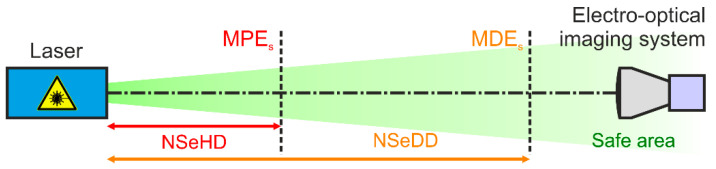
Exposure limits for sensors (Maximum Permissible Exposure for a Sensor (${MPE}_{S}$) and Maximum Dazzle Exposure for a Sensor (${MDE}_{S}$)) and corresponding hazard distances (Nominal Sensor Hazard Distance (NSeHD) and Nominal Sensor Dazzle Distance (NSeDD)).

In our earlier work, we defined equivalent quantities for electro-optical imaging systems, as illustrated in Figure 1:

- Maximum Permissible Exposure for a Sensor, ${MPE}_{S}$: The maximum applicable laser irradiance at the entrance aperture of the camera lens to prevent sensor damage.
- Nominal Sensor Hazard Distance, NSeHD: The hazard distance corresponding to the ${MPE}_{S}$.
- Maximum Dazzle Exposure for a Sensor, ${MDE}_{S}$: The laser irradiance at the entrance aperture of the camera lens that corresponds to a certain dazzle level (see definition in Section 2.4.2).
- Nominal Sensor Dazzle Distance, NSeDD: The hazard distance corresponding to the ${MDE}_{S}$.

While our earlier work was focused on the exposure limits, ${MPE}_{S}$ and ${MDE}_{S}$ [17], this publication is dedicated to the hazard distances which result from these exposure limits. Initially, we thought that the hazard distances could be calculated from the exposure limits using the well-known equations for laser eye safety (see Section 3). However, we recognized that the equations for the exposure limits lead to an unexpected dependency: The exposure limits for electro-optical imaging systems depend on the diameter of the laser beam at the camera lens and thus on the distance between laser source and sensor. This is in contrast to the human eye, where such a dependence does not occur or was not considered for simplicity. This dependence on distance leads to interesting effects regarding the hazard distances, NSeHD and NSeDD, which we would like to discuss in this publication. Furthermore, we established suitable closed-form approximations to estimate the NSeHD and the NSeDD values.

In Section 2, we first review our derivation of the sensor exposure limits. Then, in Section 3, we discuss, in particular, the implications of our equations for the exposure limits on the resulting hazard distances. Furthermore, we derive approximative equations in closed form for the hazard distances. In Section 4, we investigate the accuracy of these approximative equations in more detail.

Before doing so, however, we would like to make a comment on the terminology we use in this publication. We use the term *electro-optical imaging system* (or shortened *imaging system*) to refer to a system consisting of a *camera lens* and a *camera*. An essential component of the camera is the *image sensor* (or *imaging sensor* or *detector*), which enables the generation of electronic information (the digital image) from the incident photons. However, it may happen that we sometimes refer to the electro-optical imaging system simply as a *sensor*, very noticeable, for example, in the term *Maximum Permissible Exposure for a Sensor*, which, of course, refers to an electro-optical imaging system. The reader may forgive us for this.

## 2. Exposure Limits for Electro-Optical Imaging Systems

Whether damage or dazzle occurs or not when an image sensor is irradiated by laser light depends on the characteristics of the image sensor, as well as on the spatial distribution of the laser light at the image sensor. Here, we summarize our earlier work regarding the derivation of equations to estimate exposure limits for electro-optical imaging systems [17]. This includes a theoretical model for the estimation of the irradiance distribution at the focal plane of camera lenses. A more detailed summary for this theoretical model can also be found in Reference [18]. For all the theoretical work both in our earlier and current publication, we tried to satisfy the following objectives:

- Objective 1. Equivalent to laser safety calculations for the human eye, the values of ${MPE}_{S}$ and ${MDE}_{S}$ are stated at the position of the entrance aperture of the camera lens.
- Objective 2. The equations for the laser safety quantities are given as closed-form expressions containing only commonly known operations and functions. The equations should be as simple as possible but still sufficiently accurate.
- Objective 3: The equations for the laser safety quantities should contain—as far as it is practical to do so—only the standard parameters of the involved devices (laser, camera lens, and image sensor/camera), as specified by the manufacturer and the underlying scenario (e.g., distance and atmospheric extinction).

Comment on the applicability of our work: Above all, our laser safety calculations for electro-optical imaging systems were developed for the visible spectral range in mind. Some preliminary performed tests indicate their usability also for the near- and shortwave infrared. For the mid-wave or long-wave infrared, we cannot assure validity.

### 2.1. Parameters

Table 1 lists all parameters that we use for our laser safety calculations. According to Objective 3, our equations are based only on standard parameters that are typically specified by the manufacturers of lasers, camera lenses, or image sensors. However, some additional parameters are required, which are usually not specified or known:

- The damage threshold of an image sensor, $E_{dam}$.
- The saturation threshold of an image sensor, $E_{sat}$.
- The scatter parameters of a camera lens: s, $b_{0}$, and l (see Section 2.3.1).

In Appendix A, we state values/equations for these non-standard parameters that may be used, if measured values for a specific electro-optical imaging system are not available.

### 2.2. Scenario

**Figure 2:**
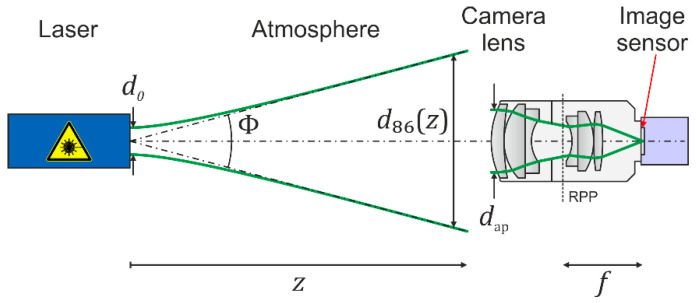
Schematic view of a dazzle scenario. RPP: rear principal plane. Please note that the location and size of the apertures and pupils are drawn for illustrative purposes only.

We assume a scenario as depicted in Figure 2. A laser emits a beam with a Gaussian beam profile characterized by output power, $P_{0}$; wavelength, λ; output beam diameter, $d_{0}$; and full angle divergence, Φ, which illuminates an electro-optical imaging system at a distance, z, consisting of a camera lens and a camera with an image sensor. The laser beam diameter at the camera lens is denoted by $d_{86}$ or $d_{63}$, depending on whether the irradiance refers to the 1/e² or 1/e points of the peak irradiance, respectively. These two quantities are related by the following:

(1) $$d_{86}=\sqrt{2}\cdot d_{63}.$$

The camera lens is described by the focal length, f, and the diameter of the entrance pupil, $d_{ap}$. We would like to point out that the location and size of the entrance pupil and the rear principal plane (RPP), as well as the beam paths, in Figure 2 are drawn for illustrative purpose only. Further parameters of the camera lens are the f-number, $F=f/d_{ap}$; the number of optical elements, $N_{oe}$; the transmittance, T; and the scatter parameters, s, $b_{0}$, and l.

The ratio of the beam diameter ($d_{86}$) to the diameter of the camera lens’ entrance pupil ($d_{ap}$) is called the truncation factor (ν) and has a determining influence on the distribution of the laser light in the focal plane of the camera lens:

(2) $$\nu =d_{86}/d_{ap}$$

Using the truncation factor (ν), the fraction ($P_{in}$) of the laser power ($P_{0}$) that enters the lens can be calculated by the following equation:

(3) $$P_{in}=P_{0}\left(1-\exp\left(-\frac{2}{\nu^{2}}\right)\right).$$

The attenuation of the laser power by the atmosphere may be included by replacing $P_{0}$ with $P_{0}\cdot \exp\left(-\mu z\right)$, where μ is the atmospheric extinction coefficient. Furthermore, atmospheric turbulence may be included using the approach of Özbilgin et al. that considers an increase in the beam diameter, $d_{86}$, due to long-term beam spread and beam wander [19].

In our theoretical model, the incident power ($P_{in}$) contributes to the focal plane irradiance distribution ($E_{fp}$) through two components: (a) the scatter/stray light component, $E_{s}$, and (b) the diffraction component, $E_{d}$, which are described in the following Section 2.3.

### 2.3. Estimation of the Focal Plane Irradiance Distribution

#### 2.3.1. Stray Light Component

**Figure 3:**
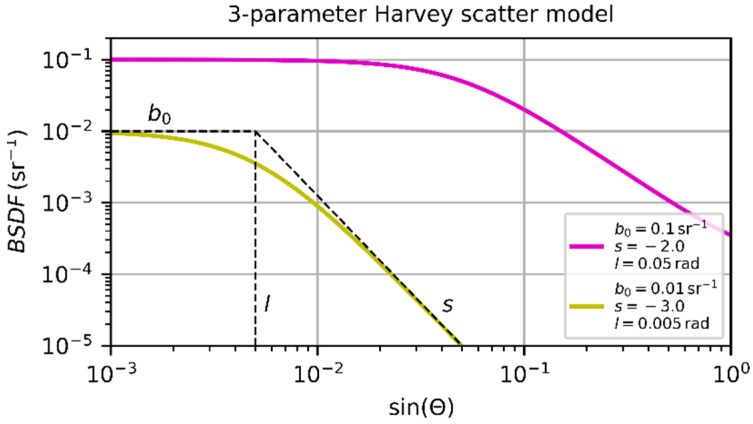
Bidirectional scattering distribution function according to the three-parameter Harvey scatter model for two different sets of scatter parameters.

To estimate the contribution of the stray light, $E_{s}$, to the focal plane irradiance distribution, we rely on the work of G. L. Peterson, who published an analytical approach for this task [20], using the three-parameter Harvey scatter model as bidirectional scattering distribution function (BSDF). This type of BSDF describes the angular distribution of stray light caused by scatter from smooth surfaces of optical elements, using three parameters: s, $b_{0}$, and l. Other sources of stray light, such as multiple reflections or scatter from the lens housing, are not considered. For a detailed explanation of the BSDF and the meaning of the scatter parameters, we refer the reader to other publications, e.g., Reference [21]. A short explanation is presented in Figure 3: Scatter parameter $b_{0}$ describes the maximum BSDF value for a scatter angle, Θ=0. Scatter parameter l indicates the scatter angle where the BSDF changes from a rather constant region (Θ<l) to a decreasing region (Θ>l). For scatter angles Θ≫l, the scatter parameter s represents the slope of the BSDF in a double-logarithmic plot.

For our theoretical model, we applied some simplifications to the work of Peterson in order to keep the equations manageable for typical camera lenses with five or more optical elements, $N_{oe}\geq 5$. We calculate the stray light component by using the following equation [17]:

(4) $$E_{s}\left(r\right)=\frac{P_{0}TN_{ss}b_{0}}{f^{2}{\left(v^{*}\right)}^{2}}{ [1+{\left(\frac{r}{v^{*}lf}\right)}^{2}]}^{\frac{s}{2}}\cdot \left(1-\exp\left(-\frac{2}{\nu^{2}}\right)\right)$$

where $v^{*}$ is defined by [17];

(5) $$\nu^{*}=\min\left(1,\frac{\nu }{\sqrt{2}}\right).$$

where $N_{ss}$ is the number of scattering surfaces of the camera lens, which we assume is twice the number of optical elements, $N_{oe}$. The modified truncation factor, $\nu^{*}$, considers that the beam diameter within the camera lens cannot increase if a laser beam, which is already much larger than the entrance aperture, would expand further, e.g., with increasing distance to the laser source.

#### 2.3.2. Diffraction Component

For the diffraction component, we assume in our theoretical model a Gaussian beam and thus apply the diffraction pattern of a truncated Gaussian beam for our calculations, e.g., see Reference [22]. The form of this diffraction pattern depends on the value of the truncation factor, ν, and consists of a central lobe that can be approximated by a Gaussian distribution and diffraction rings of lower power similar to the Airy diffraction pattern.

The central lobe is approximated by using [17]

(6) $$E_{cl}\left(r\right)=E_{0}(\nu )\exp\left(-8\frac{r^{2}}{d_{spot}^{2}}\right)$$

where the peak irradiance of the diffraction pattern is given by [23]

(7) $$E_{0}\left(\nu \right)=\frac{P_{0}T\pi }{4\lambda^{2}F^{2}}\cdot {2\nu }^{2}{ [1-\exp\left(-\frac{1}{\nu^{2}}\right)]}^{2}$$

and the spatial extent of the central lobe is calculated by [22]

(8) $$d_{spot}=k\lambda F.$$

Here, k is a spot size constant, which also depends on the truncation factor, ν; see Reference [22] for details. Outside the central lobe, the wings of the diffraction pattern are described by the mean of the diffraction ring irradiance, which is given by the following [22]:

(9) $$E_{dr}\left(r\right)=\frac{P_{0}T\lambda F}{\pi^{3}r^{3}}\cdot \frac{2}{\nu^{2}}exp\left(-\frac{2}{\nu^{2}}\right)$$

Since Equation (9) describes the mean of the diffraction ring irradiance, there is no oscillating term in the formula. Here, the diffraction ring irradiance, $E_{dr}$, is approximated by a monotonically decreasing function with a 1/r^3^ dependence.

Please note: In the equations above, as well as in all subsequent equations, the dependency on the radial coordinate, r (in the focal plane), can be replaced by the dependency on the viewing angle, Θ, using the relationship

(10) $$\Theta =\frac{r}{f}\;\;⟺\;\;r=\Theta f.$$

### 2.4. Exposure Limits

#### 2.4.1. Maximum Permissible Exposure for a Sensor (${MPE}_{S}$)

The Maximum Permissible Exposure for a Sensor ${MPE}_{S}$ can be calculated by equalizing the focal plane peak irradiance, $E_{0}(\nu )$, of Equation (7) and the image sensor’s damage threshold, $E_{dam}$. This leads to

(11) $$E_{0}\left(\nu \right)=\frac{P_{max}T\pi }{4\lambda^{2}F^{2}}\cdot {2\nu }^{2}{ [1-\exp\left(-\frac{1}{\nu^{2}}\right)]}^{2}≝E_{dam}$$

Resolving for the maximum permissible laser power, $P_{max}$, and calculating the corresponding laser peak irradiance by dividing $P_{max}$ by $\frac{\pi }{4}d_{63}^{2}=\frac{\pi }{8}d_{86}^{2}=\frac{\pi }{8}{\left(\nu \frac{f}{F}\right)}^{2}$ results in the wanted ${MPE}_{S}$ [17]:

(12) $${MPE}_{S}=E_{dam}\cdot \frac{16\lambda^{2}F^{4}}{T\pi^{2}f^{2}}{ [\frac{\frac{1}{\nu^{2}}}{1-\exp\left(-\frac{1}{\nu^{2}}\right)}]}^{2}$$

The lowest value of the ${MPE}_{S}$ (worst case) occurs for ν→∞ [17]:

(13) $${MPE}_{S,min}=\underset{\nu \to \infty }{\lim}{MPE}_{S}=E_{dam}\cdot \frac{16\lambda^{2}F^{4}}{T\pi^{2}f^{2}}$$

Equation (13) is applicable when the laser source is far away from the imaging system and the laser beam overfills the aperture of the camera lens. In this case, no distance dependence exists.

#### 2.4.2. Maximum Dazzle Exposure for a Sensor (${MDE}_{S}$)

For the human eye, the maximum dazzle exposure is not just a single value but is stated for specific dazzle levels [16]. These dazzle levels range from very low to low, medium, and high, which corresponds to angular dazzle fields of 2°, 10°, 20°, and 40°, respectively.

**Figure 4:**
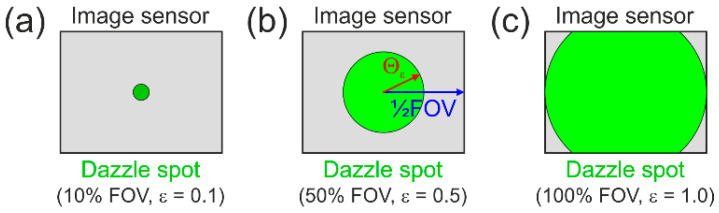
Definition of dazzle levels as fraction of the imaging system’s field of view that is dazzled: (**a**) dazzle level of ϵ=0.1, (**b**) dazzle level of ϵ=0.5, and (**c**) dazzle level of ϵ=1.0.

For electro-optical imaging systems, an equivalent definition of such default values for the dazzle field is not useful since the system’s field of view (FOV) changes with the focal length of the camera lens. Thus, we define the dazzle level as the fraction, ϵ, of the system’s FOV that is dazzled. This means, e.g., that for an incident irradiance of ${MDE}_{S}\left(\epsilon =0.1\right)$, ${MDE}_{S}(\epsilon =0.5)$, and ${MDE}_{S}\left(\epsilon =1.0\right)$, a tenth of the FOV, half of the FOV, and the full FOV is dazzled, respectively. Figure 4 illustrates that approach. The fraction, ϵ, shall be understood as the diameter of the dazzle spot divided by the size of the longer side of the image sensor.

Using this definition, the angular radius of the dazzle spot is

(14) $$\Theta_{\epsilon }=\epsilon \cdot \frac{FOV}{2},$$

where the field of view of the imaging system can be calculated by

(15) $$FOV=\frac{N_{max}\cdot p}{f}\;\;\;with\;\;\;N_{max}=\max\left(N_{col},N_{row}\right).$$

We can find the ${MDE}_{S}$ for a specific dazzle level, ϵ, by equating the focal plane irradiance distribution, $E_{fp}(\Theta_{\epsilon })$, and the image sensor’s saturation threshold, $E_{sat}$. In the case of reasonable laser dazzle, i.e., a larger amount of the system’s field of view is affected by laser radiation, the central lobe of the diffraction pattern has no influence on the spatial extent of the dazzle spot, since its diameter is typically in the order of only some sensor pixels. Thus, the focal plane irradiance distribution, $E_{fp}(\Theta_{\epsilon })$, can be approximated by the diffraction ring irradiance, $E_{dr}\left(r\right)$, and the stray light component, $E_{s}\left(r\right)$:

(16) $$E_{fp}\left(r=\Theta_{\epsilon }f\right)\approx E_{dr}\left(\Theta_{\epsilon }f\right)+E_{s}\left(\Theta_{\epsilon }f\right)≝E_{sat}\;\;\;\Leftrightarrow \;\frac{P_{max}T\lambda F}{\pi^{3}\Theta_{\epsilon }^{3}f^{3}}\cdot \frac{2}{\nu^{2}}exp\left(-\frac{2}{\nu^{2}}\right)+\frac{P_{max}TN_{ss}b_{0}}{f^{2}{\left(v^{*}\right)}^{2}}{ [1+{\left(\frac{\Theta_{\epsilon }}{v^{*}l}\right)}^{2}]}^{\frac{s}{2}}\cdot \left(1-\exp\left(-\frac{2}{\nu^{2}}\right)\right)≝E_{sat}$$

Equivalent to the ${MPE}_{S}$, we can derive the searched quantity, ${MDE}_{S}$, by resolving for the maximum permissible laser power, $P_{max}$, and calculating the corresponding laser irradiance by dividing $P_{max}$ by $\frac{\pi }{4}d_{63}^{2}=\frac{\pi }{8}d_{86}^{2}=\frac{\pi }{8}{\left(\nu \frac{f}{F}\right)}^{2}$ [17]:

(17) $${MDE}_{S}\left(\epsilon \right)=\frac{4E_{sat}F^{2}}{\pi T}\frac{1}{\frac{\lambda F}{\pi^{3}f\Theta_{\epsilon }^{3}}\cdot \exp\left(-\frac{2}{\nu^{2}}\right)+\frac{N_{ss}b_{0}}{{\left(v^{*}\right)}^{2}}{ [1+{\left(\frac{\Theta_{\epsilon }}{v^{*}l}\right)}^{2}]}^{\frac{s}{2}}\cdot \frac{\left(1-\exp\left(-\frac{2}{\nu^{2}}\right)\right)}{2/\nu^{2}}}$$

As in the case of the ${MPE}_{S}$, the equation simplifies for the case of ν→∞, which gives the minimum value of the ${MDE}_{S}$ [17]:

(18) $${MDE}_{S,min}\left(\epsilon \right)=\underset{\nu \to \infty }{\lim}{MDE}_{S}(\epsilon )=\frac{4E_{sat}F^{2}}{\pi T}\frac{1}{\frac{\lambda F}{\pi^{3}f\Theta_{\epsilon }^{3}}+N_{ss}b_{0}{ [1+{\left(\frac{\Theta_{\epsilon }}{l}\right)}^{2}]}^{\frac{s}{2}}}$$

Equations (17) and (18) should not be used for vanishing values of ϵ, such as ϵ=0 or ϵ very close to zero; see Reference [17] for more details. The value ${MDE}_{S}(\epsilon =0)$ would correspond to the onset of laser dazzle. This onset can be estimated by using Equations (12) or (13) but replacing the damage threshold, $E_{dam}$, by the saturation threshold, $E_{sat}$ [17]:

(19) $${MDE}_{S}(\epsilon =0)=E_{sat}\cdot \frac{16\lambda^{2}F^{4}}{T\pi^{2}f^{2}}{ [\frac{\frac{1}{\nu^{2}}}{1-\exp\left(-\frac{1}{\nu^{2}}\right)}]}^{2}$$

(20) $${MDE}_{S,min}\left(\epsilon =0\right)=\underset{\nu \to \infty }{\lim}{MDE}_{S}(\epsilon =0)=E_{sat}\cdot \frac{16\lambda^{2}F^{4}}{T\pi^{2}f^{2}}$$

## 3. Laser Hazard Distances

Using the equations for the estimation of exposure limits for electro-optical imaging systems according to Section 2.4.1 (damage) and Section 2.4.2 (dazzle), we can now take a closer look at the corresponding hazard distances. The calculation of the hazard distances for sensors (NSeHD and NSeDD) following the classical laser safety standards related to the human eye would be as follows [10,11]:

(21) $$NSeHD=\frac{\sqrt{\frac{4P_{0}}{\pi \cdot MPE_{s}}-d_{0}^{2}}}{\Phi }\approx \frac{\sqrt{\frac{4P_{0}}{\pi \cdot MPE_{s}}}-d_{0}}{\Phi }$$

(22) $$NSeDD=\frac{\sqrt{\frac{4P_{0}}{\pi \cdot MDE_{s}}-d_{0}^{2}}}{\Phi }\approx \frac{\sqrt{\frac{4P_{0}}{\pi \cdot MDE_{s}}}-d_{0}}{\Phi }$$

In each case, the first term given in Equations (21) and (22) corresponds to ANSI Z136.6 [10] and uses the following equation to calculate the laser beam diameter: $d(z)=\sqrt{d_{0}^{2}+\Phi^{2}z^{2}}$. The second term is an approximation utilizing a simplified calculation of the beam diameter: $d\left(z\right)=d_{0}+\Phi z,$ as used in the German Technical Rules regarding the Occupational Health and Safety Ordinance on Artificial Optical Radiation [11].

As a side note, Equations (21) and (22) require the beam diameter, $d_{0}$, and the beam divergence, Φ, related to the 1/e-intensity points of the beam profile, according to the rules of occupational laser safety standards. When using the 1/e²-definition for beam diameter and divergence, Equations (21) and (22) have to be modified by replacing the factor 4 by the factor 8.

Unfortunately, the calculation of hazard distances for imaging systems is different. As we can see from Equations (12) and (17), both the ${MPE}_{S}$ and the ${MDE}_{S}$ depend on the truncation factor, ν. This implicates a dependence of the exposure limit on the distance between laser source and imaging system since the beam diameter changes with distance and, thus, so does the truncation factor.

Let us conduct a thought experiment: An imaging system is located far away from a laser source (ν→∞). The operator of the system calculates, for example, the ${MPE}_{S}$, which is given in this case by Equation (12) and, subsequently, the corresponding hazard distance NSeHD by using Equation (21). Then, the operator moves the system closer to the laser source but only until he reaches the calculated hazard distance, because he wants to preserve the system from laser damage. If we assume a typical laser beam, which is collimated or slightly divergent, the size of the laser beam will be smaller at this position as compared to the starting position. Thus, the truncation factor, ν, will be decreased, and, according to Equation (12), the ${MPE}_{S}$ will increase! Calculating again the NSeHD using this increased value of ${MPE}_{S}$ will now result in some lower value of the hazard distance. This means that the operator can further approach the laser source without danger for the imaging system. Then, the truncation factor will again be decreased, and, thus, the same applies to the hazard distance. This is the laser safety equivalent to Zeno’s paradox of Achilles and the tortoise. The ${MDE}_{S}$ behaves in a similar manner.

**Figure 5:**
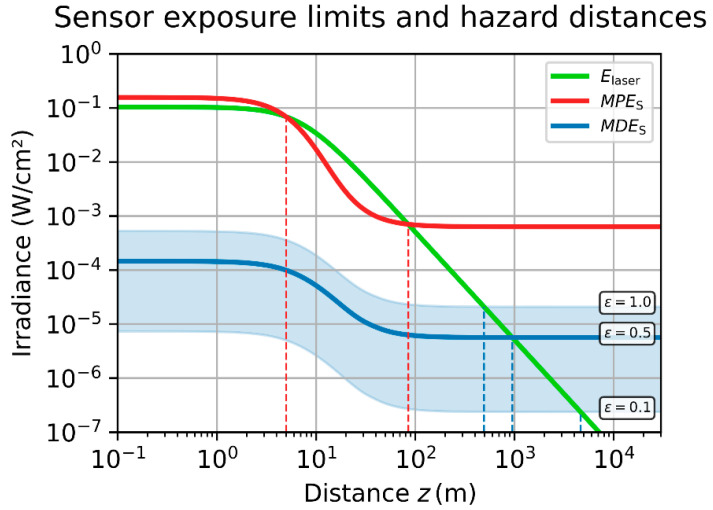
Example of ${MPE}_{S}$ (red line) and ${MDE}_{S}$ (light blue band corresponding to various values of dazzle level, ϵ) for an arbitrarily chosen scenario as a function of distance between imaging system and laser source. Additionally, the peak irradiance of the assumed laser source at the position of the lens is shown (green line). The parameters used for the calculations are listed in Table 2.

The distance-dependent values of ${MPE}_{S}$ and ${MDE}_{S}$ are plotted in Figure 5 for a generic electro-optical imaging system and laser source; the parameters assumed for this calculation are listed in Table 2. While the ${MPE}_{S}$ is a single curve (plotted in red), the ${MDE}_{S}$ is represented by a light blue band, which indicates the range of ${MDE}_{S}$ values for various dazzle levels, $\epsilon \in [0.1;1.0]$. The ${MDE}_{S}$ curve for ϵ=0.5 is highlighted by a blue line; the upper and lower border of blue band correspond to ϵ=1.0 and ϵ=0.1, respectively. Additionally, the laser peak irradiance at the front-face of the camera lens is shown as a green curve. Please note that the parameter $E_{dam}$ in Table 2 is related to the focal plane, whereas the ordinate of the plot is related to the front-face of the camera lens.

In Figure 5, we can recognize how the ${MPE}_{S}$ and ${MDE}_{S}$ vary with distance. Following the curves starting from large distance values, the exposure limits are quite constant, which correspond to the minimum values as given by Equations (13) and (18). For closer distances from about ~40 m to ~4 m, the exposure limits increase strongly (for the given example) with the decreasing distance. Finally, for distances below ~1 m, the exposure limits stay constant again.

A very important result concerns the hazard distance regarding the ${MPE}_{S}$ curve: Looking at the green curve, which indicates the laser peak irradiance, we can see that this curve intersects the ${MPE}_{S}$ curve twice. As long as the irradiance curve is below the ${MPE}_{S}$ curve, the imaging system is safe from damage. In the distance range where the irradiance curve is above the ${MPE}_{S}$ curve, the imaging system is not safe from damage. By the example of Figure 5, we can recognize two intersection points, $\nu_{hd}$, which cut the ${MPE}_{S}$ curve into three sections: In the first section, the image sensor is safe from damage within the distance from the laser to the first intersection point. Between the two intersection points, the image sensor is not safe from damage, and in section three, beyond the second intersection point, the image sensor is again safe from damage. This means that the laser hazard zone for imaging systems may have a distance dependent upper, as well as a lower limit. This is in contrast to laser hazard distances that are valid to the human eye.

In order to explain this behavior, it is important to consider that the focal spot size of a truncated Gaussian beam increases with the decreasing truncation factor, ν; see, for example, Reference [22]. Starting at large distances, the diameter of the laser beam, $d_{86}$, is much larger than the diameter of the lens aperture, $d_{ap}$ (more precisely: entrance pupil). Thus, only a low portion of the laser power, $P_{0}$, enters the lens. Although the laser spot size, $d_{spot}$, at the focal plane is the minimum (Airy diffraction pattern) and the focal plane irradiance, $E_{fp}$, is below the damage threshold, $E_{dam}$, of the image sensor, the imaging system is safe from damage. As the imaging system moves closer to the laser source, the beam diameter at the lens aperture becomes smaller and smaller, and, thus, more and more power enters the lens. At a certain distance, the incident power, $P_{in}$, is that high that the focal plane irradiance reaches the damage threshold of the image sensor and damage occurs. This distance corresponds to the hazard distance, NSeHD. However, when the laser beam size is comparable or lower than the diameter of the lens aperture ($d_{86}\leq d_{ap})$, we come into the regime of a truncated Gaussian beam. When the truncation factor becomes very low, there is a considerable increase in focal spot size that reduces the focal plane irradiance, $E_{fp}$, to such an extent that it falls below the damage threshold, $E_{dam}$.

We have to admit that we intentionally chose the laser parameters in such a way that this effect becomes eye-catching in the example of Figure 5. If we chose a sufficiently higher value of laser power, $P_{0}$, the irradiance curve would be shifted so much upwards that the irradiance curve would intersect the ${MPE}_{S}$ curve only once. In our example, this is the case for the ${MDE}_{S}$ curves in Figure 5. For each dazzle level, ϵ (0.1, 0.5 or 1.0), the irradiance curve will intersect a specific ${MDE}_{S}$ curve only once. For another choice of system parameters (mainly the scatter parameter, s), that may be different.

At this point at least, it is clear that we cannot simply apply Equations (21) and (22) to the ${MPE}_{S}$ and ${MDE}_{S}$ values, as given by Equations (12) and (17), respectively, in order to calculate the corresponding hazard distances. The question is now, how can we calculate hazard distances for imaging systems when assuming a truncated Gaussian laser beam?

We started the search for this answer by using the example of the NSeHD in Section 3.1. Subsequently, we discuss the more complicated case of the NSeDD in Section 3.2. As a side note, Equations (21) and (22) can be used without limitation to estimate a maximum value of NSeHD and NSeDD by using the minimum values of the ${MPE}_{S}$ and ${MDE}_{S}$ as given by Equations (13) and (18), since these quantities do not depend on the truncation factor. This corresponds to the worst-case scenario.

Remark: The above-described effect of an increasing focal plane spot size due to a decreasing laser beam diameter is not considered for laser eye safety. According to Schulmeister, “it has to be kept in mind that for a collimated laser beam the retinal spot will always be minimal, irrespective of the beam diameter at the cornea” [24], and “in terms of biophysical processes, it is not actually the irradiance at the cornea that is the relevant quantity, but rather the power that enters the eye through the pupil and that is incident on the retina.” Thus, in laser eye safety, the concept of the limiting aperture is used to calculate irradiance values from power measurements in order to compare these irradiance values with the exposure limit.

### 3.1. Nominal Sensor Hazard Distance (NSeHD)

The hazard distance NSeHD is defined by the fact that the laser irradiance at the system’s camera lens is equal to the ${MPE}_{S}$: $E_{laser}\left(z=NSeHD\right)≝{MPE}_{S}$. The laser peak irradiance is given by

(23) $$E_{laser}\left(z\right)=\frac{8P_{0}}{\pi d_{86}^{2}},$$

where the laser beam diameter, $d_{86}$, depends on the distance, z, between the imaging system and laser source:

(24) $$d_{86}(z)=\sqrt{d_{0}^{2}+\phi^{2}z^{2}}$$

However, since the truncation factor, ν, is the determining parameter for the ${MPE}_{S}$, we stick to this quantity and rewrite Equation (23) by using the relations $d_{86}=\nu \cdot d_{ap}\;$ and $d_{ap}=f/F$, leading to the following:

(25) $$E_{laser}\left(\nu \right)=\frac{8P_{0}F^{2}}{\pi \nu^{2}f^{2}}.$$

**Figure 6:**
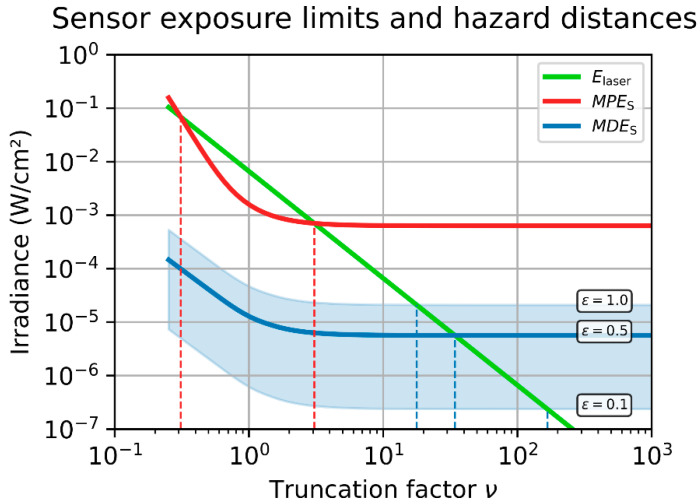
Example of ${MPE}_{S}$ (red line) and ${MDE}_{S}$ (light blue band corresponding to various values of dazzle level, ϵ) for an arbitrarily chosen scenario as a function of the truncation factor. Additionally, the peak irradiance of the assumed laser source is plotted (green line). The parameters used for the calculations are listed in Table 2.

For Figure 6, we used the same parameters as for Figure 5, but the laser peak irradiance and the ${MPE}_{S}$/${MDE}_{S}$ curves are now plotted as a function of the truncation factor, ν. In the double-logarithmic plot, the laser peak irradiance (green line) is decreasing with a slope of −2. From the intersections of the ${MPE}_{S}$ curve (red line) with the laser peak irradiance (red line), we can deduce the truncation factor, $\nu_{hd}$, corresponding to the hazard distances for sensor damage.

As discussed above, depending on the laser peak irradiance, one intersection point only or two intersection points may result. Of course, in the case of very low laser power, there will exist no intersection point at all.

If there exists a $\nu_{hd}$ value, we then can calculate the corresponding hazard distance by using the relation $d_{86}=\nu \cdot d_{ap}$ and Equation (24):

(26) $$\nu_{hd}d_{ap}=\sqrt{d_{0}^{2}+\phi^{2}{NSeHD}^{2}}$$

or

(27) $$NSeHD=\frac{\sqrt{\nu_{hd}^{2}d_{ap}^{2}-d_{0}^{2}}}{\Phi }=\frac{\sqrt{\nu_{hd}^{2}\cdot f^{2}/F^{2}-d_{0}^{2}}}{\Phi }$$

To find the quantity $\nu_{hd}$, we equate Equation (12) (${MPE}_{S}$) and Equation (25) (laser irradiance at the lens) and obtain

(28) $$E_{dam}\cdot \frac{16\lambda^{2}F^{4}}{T\pi^{2}f^{2}}{ [\frac{\frac{1}{\nu_{hd}^{2}}}{1-\exp\left(-\frac{1}{\nu_{hd}^{2}}\right)}]}^{2}≝E_{laser}\left(\nu_{hd}\right)=\frac{8P_{0}F^{2}}{\pi \nu_{hd}^{2}f^{2}}.$$

Equation (28) can be rewritten as

(29) $$f(\nu_{hd})≝K$$

with

(30) $$f\left(\nu \right)=\frac{1}{\nu^{2}{ [1-\exp\left(-\frac{1}{\nu^{2}}\right)]}^{2}},\;K=\frac{P_{0}T\pi }{2E_{dam}\lambda^{2}F^{2}}.$$

In Equation (29), only the term $f(\nu_{hd})$ on the left-hand side depends on $\nu_{hd}$, whereas the term on the right-hand side is a constant.

Additionally, we define two basic terms, $b_{1}\left(\nu \right)$ and $b_{2}\left(\nu \right)$, from which f(ν) is composited:

(31) $$f\left(\nu \right)=\frac{1}{\nu^{2}{ [1-\exp\left(-\frac{1}{\nu^{2}}\right)]}^{2}}=b_{1}\left(\nu \right)\cdot { [b_{2}\left(\nu \right)]}^{2},$$

with

(32) $$b_{1}\left(\nu \right)=\frac{1}{\nu^{2}}\;\;\;and\;\;\;b_{2}\left(\nu \right)=\frac{1}{1-\exp\left(-\frac{1}{\nu^{2}}\right)}.$$

**Figure 7:**
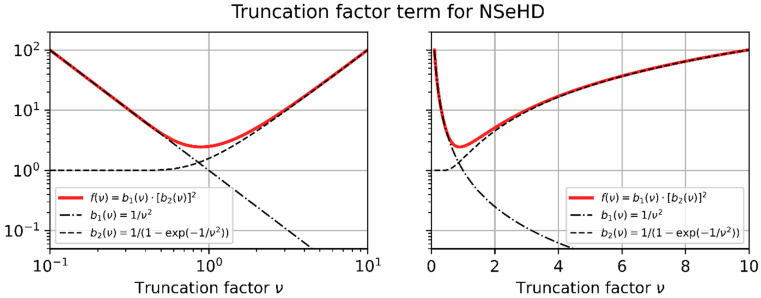
Truncation factor term, f(ν), which is related to the NSeHD. The function, f(ν), is built up by the functions $b_{1}(\nu )$ and $b_{2}(\nu )$. Left, logarithmic horizontal axis; right, linear horizontal axis.

In Figure 7, we plotted f(ν), $b_{1}\left(\nu \right)$, and $b_{2}\left(\nu \right)$ in two graphs: in the graph on the left-hand side, using a logarithmic horizontal axis; and in the graph on the right-hand side, using a linear horizontal axis. The truncation factor term, $f\left(\nu \right)$, is plotted as a red line, whereas the basic terms, $b_{1}\left(\nu \right)$ and $b_{2}\left(\nu \right)$, are shown as black lines.

From the graphs of Figure 7, we can deduce that Equation (29) will have either two solutions ($K>\min\left(f\left(\nu \right)\right)$, one solution ($K=\min\left(f\left(\nu \right)\right)$, or no solution ($K<\min\left(f\left(\nu \right)\right)$. Unfortunately, there is no analytical solution for Equation (29), only a numerical one. However, fortunately, various closed-form approximations for $\nu_{hd}$ are possible.

#### 3.1.1. A Simple Approach to Estimate the NSeHD

A very simple approximation for $\nu_{hd}$ is obtained by solving Equation (29) for each of the two basic terms:

(33) $$b_{1}\left(\nu_{hd}\right)=\frac{1}{\nu_{hd}^{2}}≝K$$

and

(34) $$b_{2}\left(\nu_{hd}\right)=\frac{1}{1-\exp\left(-\frac{1}{\nu_{hd}^{2}}\right)}≝K.$$

The solution of Equation (33) delivers an approximate-value $\nu_{hd}$ for the lower hazard distance (abbreviation: lhd), and the solution of Equation (34) delivers an approximate-value $\nu_{hd}$ for the upper hazard distance (abbreviation: uhd):

(35) $$\nu_{lhd}^{2}=\frac{1}{K}=\frac{2E_{dam}\lambda^{2}F^{2}}{P_{0}T\pi }$$

(36) $$\nu_{uhd}^{2}=\frac{1}{\ln\left(\frac{K}{K-1}\right)}=\frac{-1}{\ln\left(1-K\right)}=\frac{-1}{\ln\left(1-\frac{P_{0}T\pi }{2E_{dam}\lambda^{2}F^{2}}\right)}$$

From Figure 7, we can easily recognize that this simple approach will not be very exact in the vicinity of the minimum of f(ν). Furthermore, at least Equation (35) will always deliver a solution, although the value of the constant K may be lower than the minimum of f(ν). This means that care has to be taken when these equations are applied.

In order to apply Equations (35) and (36), one has to make sure that the constant K is equal or larger than the minimum of f(ν). The minimum of $f\left(\nu \right)$ occurs for ν≈0.892, which leads to the following constraint:

(37) $$K\geq f\left(0.892\right)\;\Leftrightarrow \;\frac{P_{0}T\pi }{2E_{dam}\lambda^{2}F^{2}}\geq 2.455$$

When this constraint is fulfilled, the NSeHD can roughly be estimated by Equation (27) by using the values derived from Equations (35) and (36). If the constraint of Equation (37) is not fulfilled, the sensor is safe from damage.

#### 3.1.2. An Improved Approach to Estimate the NSeHD 

An improved approach to solve Equation (29) is to approximate $f\left(\nu \right)$ by a simpler function, which can be resolved for ν. In a first step, $f\left(\nu \right)$ can be simplified by using the basic functions $b_{1}\left(\nu \right)$ and $b_{2}\left(\nu \right)$:

(38) $$F\left(\nu \right)=b_{1}\left(\nu \right)+b_{2}(\nu )=\frac{1}{\nu^{2}}+\frac{1}{1-\exp\left(-\frac{1}{\nu^{2}}\right)}$$

Although the function $F\left(\nu \right)$ is slightly simpler than $f\left(\nu \right)$, it is also not possible to solve $F\left(\nu_{hd}\right)≝K$ analytically. However, if we perform a Taylor expansion of the exponential function in Equation (38) and keep only the first two terms, we obtain

(39) $$F^{*}\left(\nu \right)=b_{1}\left(\nu \right)+\frac{1}{b_{1}\left(\nu \right)}=\frac{1}{\nu^{2}}+\nu^{2}.$$

**Figure 8:**
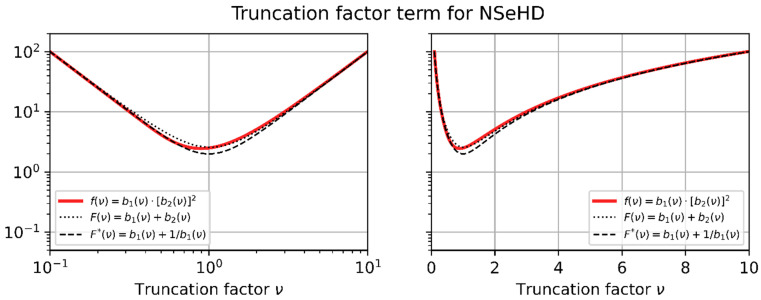
Plot of the truncation factor term, f(ν), which is related to the NSeHD. Furthermore, two approximations for f(ν) are plotted: F(ν) and $F^{*}(\nu )$. Left, logarithmic horizontal axis; right, linear horizontal axis.

The approximations $F\left(\nu \right)$ and $F^{*}\left(\nu \right)$ are plotted as black lines in the graph of Figure 8 together with the original function, $f\left(\nu \right)$ (red line).

We can now use function $F^{*}\left(\nu \right)$ for approximating $\nu_{hd}$ by solving $F^{*}\left(\nu_{hd}\right)≝K$:

(40) $$\frac{1}{v_{hd}^{2}}+v_{hd}^{2}=K\;\Leftrightarrow \;v_{hd}^{4}-Kv_{hd}^{2}+1=0,$$

which results in

(41) $$v_{uhd}^{2}=\frac{K}{2}+\sqrt{\frac{K^{2}}{4}-1}=\frac{P_{0}T\pi }{4E_{dam}\lambda^{2}F^{2}}+\sqrt{{\left(\frac{P_{0}T\pi }{4E_{dam}\lambda^{2}F^{2}}\right)}^{2}-1},\;v_{lhd}^{2}=\frac{K}{2}-\sqrt{\frac{K^{2}}{4}-1}=\frac{P_{0}T\pi }{4E_{dam}\lambda^{2}F^{2}}-\sqrt{{\left(\frac{P_{0}T\pi }{4E_{dam}\lambda^{2}F^{2}}\right)}^{2}-1}$$

for the upper and lower hazard distance values.

### 3.2. Nominal Sensor Dazzle Distance (NSeDD)

Equivalent to the NSeHD, the hazard distance, NSeDD, is defined as the distance where the laser irradiance at the imaging system’s camera lens is equal to the ${MDE}_{S}$: $E_{laser}(z=NSeDD)≝MDE_{S}$. Again, we first estimate the value of the truncation factor, $\nu_{hd}$, corresponding to the NSeDD and then, subsequently, calculate the NSeDD by using the following equation:

(42) $$NSeDD=\frac{\sqrt{\nu_{hd}^{2}d_{ap}^{2}-d_{0}^{2}}}{\Phi }=\frac{\sqrt{\nu_{hd}^{2}f^{2}/F^{2}-d_{0}^{2}}}{\Phi }.$$

However, in the case of the ${MDE}_{S}$, things are more complicated. The ${MDE}_{S}$ is given by Equation (17), which leads, together with Equation (25), to the following equation to be solved in order to obtain the $\nu_{hd}$:

(43) $${MDE}_{S}\left(\epsilon \right)≝E_{laser}\left(\nu_{hd}\right)\;\Leftrightarrow \;\;\frac{\lambda F}{\pi^{3}f\Theta_{\epsilon }^{3}}\cdot \frac{2}{\nu_{hd}^{2}}\exp\left(-\frac{2}{\nu_{hd}^{2}}\right)+\frac{N_{ss}b_{0}}{{\left(\nu_{hd}^{*}\right)}^{2}}{ [1+{\left(\frac{\Theta_{\epsilon }}{\nu_{hd}^{*}l}\right)}^{2}]}^{\frac{s}{2}}\cdot \left(1-\exp\left(-\frac{2}{\nu_{hd}^{2}}\right)\right)=\frac{E_{sat}F^{2}}{\pi T}$$

For the following discussion, we rewrite Equation (43) as follows:

(44) $$K_{1}\cdot f_{1}(\nu_{hd})+K_{2}(\nu_{hd}^{*})\cdot f_{2}(\nu_{hd})=K_{3}$$

with

(45) $$K_{1}=\frac{\lambda F}{\pi^{3}f\Theta_{\epsilon }^{3}};\;\;\;K_{2}\left(\nu^{*}\right)=\frac{N_{ss}b_{0}}{{\left(\nu^{*}\right)}^{2}}{ [1+{\left(\frac{\Theta_{\epsilon }}{\nu^{*}l}\right)}^{2}]}^{\frac{s}{2}};\;\;\;K_{3}=\frac{E_{sat}f^{2}}{P_{0}T}\;f_{1}\left(\nu \right)=\frac{2}{\nu^{2}}\exp\left(-\frac{2}{\nu^{2}}\right);\;\;\;f_{2}\left(\nu \right)=1-\exp\left(-\frac{2}{\nu^{2}}\right).$$

$K_{1}$ and $K_{3}$ are constants, which are completely independent of the truncation factor ν. For $\nu \geq \sqrt{2}$, the term $K_{2}\left(\nu^{*}\right)$ becomes independent of ν since $\nu^{*}=1$; see the definition of $\nu^{*}$ according to Equation (5).

**Figure 9:**
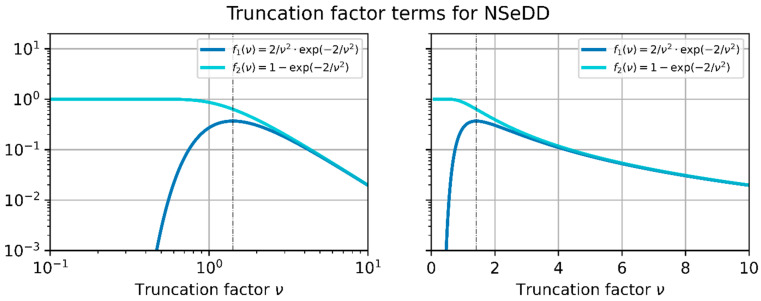
Truncation factor terms $f_{1}(\nu )$ and $f_{2}(\nu )$, which are related to the NSeHD. Left, logarithmic horizontal axis; right, linear horizontal axis.

In Figure 9, the truncation factor terms $f_{1}\left(\nu \right)$ and $f_{2}\left(\nu \right)$ are plotted as a function of ν. The dashed black vertical line marks the position at which the truncation factor is $\nu =\sqrt{2}$ and for which $f_{1}\left(\nu \right)$ reaches its maximum value.

Analogue to the NSeHD, Equation (44) cannot be solved analytically, but only numerically, to calculate the NSeDD. Thus, we tried the same approach as before: replacing those ν-dependent terms in Equation (44) by simpler functions in order to be able to resolve the modified equation for $\nu_{hd}$. Unfortunately, we could not find generally applicable approximations for the two truncation factor terms, $f_{1}(\nu )$ and $f_{2}(\nu )$, as well as for $K_{2}(\nu^{*})$, which would allow this approach to work. Nevertheless, a closer look on the left side of Equation (44) helped us provide an estimate for the NSeDD.

**Figure 10:**
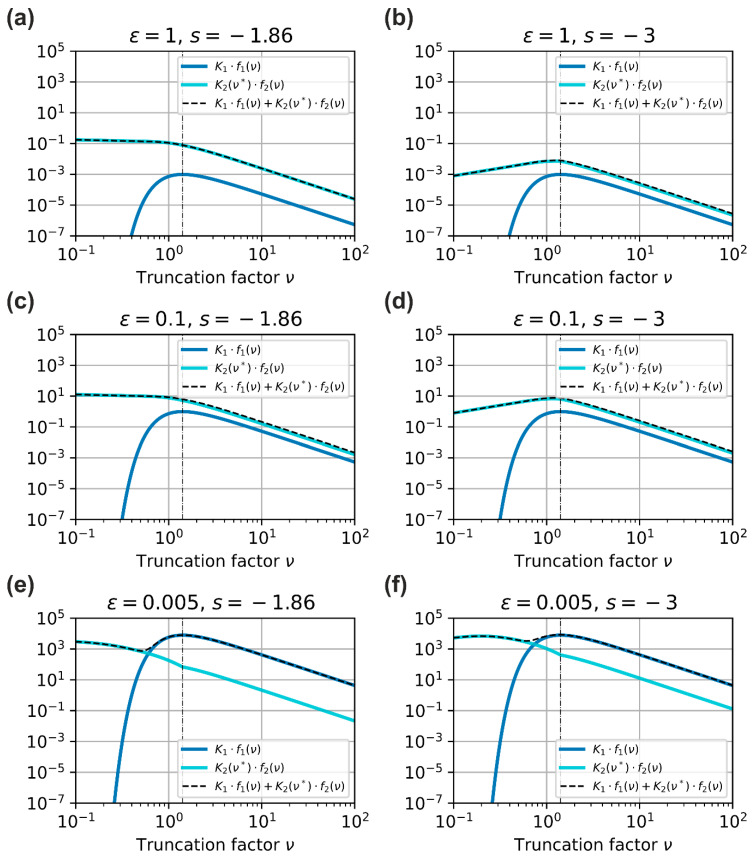
Course of the terms on the left-hand side of Equation (44) as a function of the truncation factor, ν. For the graphs, three different values of the dazzle level, ϵ (1.0, 0.1 and 0.005), and two different values of the scatter parameter, s (−1.86 and −3), were used, as stated in the title of each graph. All other parameters used for the calculations correspond to those listed in Table 2.

In Figure 10, we plotted the left-hand side of Equation (44) in dependence of the truncation factor, ν, for different values of the dazzle level, ϵ (1.0, 0.1, and 0.005), and for two different values of scatter parameter, s (−1.86 and −3). All other parameters correspond to those listed in Table 2. Again, the dashed black vertical line marks the position at which the truncation factor is $\nu =\sqrt{2}$ and for which $f_{1}\left(\nu \right)$ reaches its maximum value.

Looking at Figure 10, we can observe the following:

- For $\nu <\sqrt{2}$, the course of the left-hand side of Equation (44), $K_{1}\cdot f_{1}\left(\nu \right)+K_{2}\left(\nu^{*}\right)\cdot f_{2}\left(\nu \right)$, can be quite different depending on the parameters ϵ and s. For $\nu \geq \sqrt{2}$, the course of this term is very similar for all cases and is monotonically decreasing.
- For practically relevant values of the dazzle level, that is, ϵ>0.1, the term $K_{2}(\nu^{*})\cdot f_{2}(\nu )$ dominates, which is related to the stray light contribution.
- Only for extremely low dazzles levels, e.g., ϵ=0.005 in Figure 10e,f, the contribution related to diffraction, $K_{1}\cdot f_{1}(\nu )$, dominates due to the $1/\Theta_{\epsilon }^{3}$-dependency of factor $K_{1}$.

We can also learn from Figure 10 that Equation (44) may have no, one, or multiple solutions, $\nu_{hd}$, depending on the value of constant $K_{3}$. In the following, we will denote the solutions of Equation (44) by $\nu_{hd,i}$, where i∈[1…n] and $\nu_{hd,i}<\nu_{hd,i+1}$. Thus, the largest solution $\nu_{hd,n}$ will correspond to the upper value of the NSeDD. In the examples of Figure 10, we can see the following situations:

- Figure 10a,c: There may be no or one solution depending on constant $K_{3}$. If there is a solution $\nu_{hd}$, this solution can occur either for $\nu <\sqrt{2}$ or $\nu \geq \sqrt{2}$.
- Figure 10b,d: There may be no, one or two solutions depending on constant $K_{3}$. If there are two solutions $\nu_{hd}$, the larger one relates to upper value of the NSeDD and the lower one relates to the lower value of the NSeDD.
- Figure 10e: There may be up to three solutions depending on constant $K_{3}$.
- Figure 10f: There may be up to four solutions depending on constant $K_{3}$.

We can state that it would be extremely demanding to provide simple analytical estimates, $\nu_{hd,i}$, for all n possible solutions of Equation (44). Even if we are able to do so, it is questionable whether the effort is really worth it for practical applications, because one must keep in mind how to interpret the results.

For this, we assume a situation as depicted in Figure 10f (dazzle level ϵ=0.005) and a very low laser power, so that there would exist four solutions for Equation (44) (n=4). Furthermore, we assume that the imaging system approaches the laser device from an infinite distance. From a certain distance, the laser dazzle of the system will occur. When the imaging system reaches the distance corresponding to $\nu_{hd,4}$, the system will experience a laser dazzle corresponding to the defined dazzle level, ϵ. For distances corresponding to smaller truncation factors in the range $\left[\nu_{hd,4},\nu_{hd,3}\right]$, the dazzle level would be larger than ϵ=0.005. In the succeeding range $\left[\nu_{hd,3},\nu_{hd,2}\right]$, the imaging system would still become dazzled, but with a dazzle level below the defined value ϵ=0.005, and so on. This means that we expect that the dazzle spot increases when the imaging system approaches the laser, and starting from a certain distance, the size of the laser spot would decrease slightly even when the distance decreases. Then, at some even closer distance, the dazzle spot would increase again for the decreasing distance.

However, in a real dynamic scenario, maybe in conjunction with fluctuations due to atmospheric turbulence, such changes would be barely noticeable. Furthermore, such a complex situation as described before occurs only for practically rather uninteresting scenarios, where we investigate an extremely low dazzle level, ϵ (corresponding to dazzle spots of only a few pixels in diameter). For example, in our calculations based on the parameters of Table 2, a dazzle level of ϵ=0.005 would correspond to a dazzle spot with an overexposed area of only 4 pixels in diameter. A system operator would recognize this as a very small bright spot in the image, which would have, however, only a small influence on the perception of the overall scene. The system performance would hardly be restricted by such a low dazzle level.

Therefore, as a practical approach for laser safety calculations, we chose to derive an estimate only for the upper value of the NSeDD, which is determined by the largest value of all solutions, $\nu_{hd,i}$, of Equation (44). For this, we still have to distinguish between the situation of $\nu \geq \sqrt{2}$ and $\nu <\sqrt{2}$, since the term $K_{2}$ will lose its dependency on the truncation factor, ν, for the first case. In Section 3.2.1, we treat the case of large beam diameters ($\nu \geq \sqrt{2}$), and in the subsequent section, Section 3.2.2, the case of small beam diameters ($\nu <\sqrt{2}$). For the latter case, we restrict our investigations on larger values of the dazzle level, ϵ, i.e., to situations as the ones depicted in the graphs of Figure 10a–d.

#### 3.2.1. NSeDD Estimate for Extended Laser Beams

The first approximation for the NSeDD applies for the case that the dazzle level of interest occurs only at a certain minimum distance of the imaging system to the laser source. Here, we assume that the diameter of the laser beam is considerably larger than the entrance pupil of the lens, or, more precisely, $d_{86}\geq \sqrt{2}\cdot d_{ap}\Leftrightarrow \nu \geq \sqrt{2}$. Thus, we obtain $\nu^{*}=1$, which means that $K_{2}$ is independent of ν and is a simple constant:

(46) $$K_{2}^{>}=N_{ss}b_{0}{ [1+{\left(\frac{\Theta_{\epsilon }}{l}\right)}^{2}]}^{\frac{s}{2}}$$

In this case, Equation (44) simplifies to

(47) $$K_{1}\cdot f_{1}\left(\nu_{hd}\right)+K_{2}^{>}\cdot f_{2}\left(\nu_{hd}\right)=K_{3}.$$

Furthermore, looking at Figure 9, we can see that the course of truncation factor terms $f_{1}(\nu )$ and $f_{2}(\nu )$ proceeds quite similar for large values of the truncation factor. In order to solve Equation (47) for $\nu_{hd}$, we thus approximate both terms by using the following:

(48) $$f_{1}\left(\nu \right)\approx f_{2}\left(\nu \right)\approx F_{2}^{>}\left(\nu \right)≔\frac{2}{\nu^{2}+2}\;\;for\;\;\nu \geq \sqrt{2}.$$

**Figure 11:**
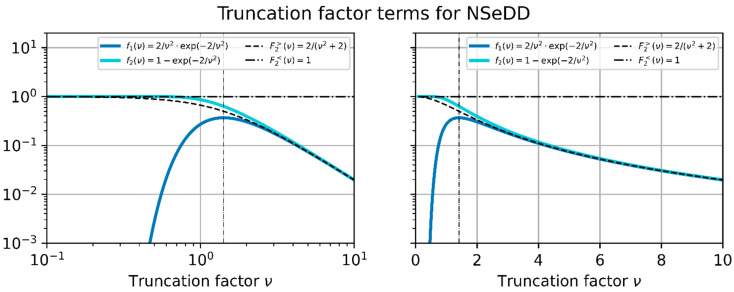
Truncation factor terms $f_{1}(\nu )$ and $f_{2}(\nu )$, which are related to the NSeHD. Furthermore, two approximations for $f_{2}(\nu )$ are plotted: $F_{2}^{>}(\nu )$ and $F_{2}^{<}(\nu )$ used for $\nu \geq \sqrt{2}$ and $\nu <\sqrt{2}$, respectively. Left, logarithmic horizontal axis; right, linear horizontal axis.

The approximation $F_{2}^{>}(\nu )$ is plotted in Figure 11, together with the truncation factor terms $f_{1}\left(\nu \right)$ and $f_{2}\left(\nu \right)$. The truncation factor $\nu \geq \sqrt{2}$ is marked by a vertical dashed line in the graphs. Basically, $F_{2}^{>}(\nu )$ is an approximation for $f_{2}\left(\nu \right)$ only but also describes the course of $f_{1}\left(\nu \right)$ for a truncation factor of $\nu \geq \sqrt{2}$ adequately.

Using approximation $F_{2}^{>}(\nu )$, Equation (47) reduces to

(49) $$\left(K_{1}+K_{2}^{>}\right)\frac{2}{\nu_{hd}^{2}+2}=K_{3}\;\Leftrightarrow \;\nu_{hd}^{2}=2\frac{K_{1}+K_{2}^{>}}{K_{3}}-2$$

The estimate for $\nu_{hd}^{2}$ is then as follows:

(50) $$\nu_{hd}^{2}=\frac{2P_{0}T}{E_{sat}f^{2}}\left(\frac{\lambda F}{\pi^{3}f\Theta_{\epsilon }^{3}}+N_{ss}b_{0}{ [1+{\left(\frac{\Theta_{\epsilon }}{l}\right)}^{2}]}^{\frac{s}{2}}\right)-2$$

The NSeDD can subsequently be calculated using Equation (42). As mentioned before, for $\nu \geq \sqrt{2}$, the functions $f_{1}\left(\nu \right)$ and $f_{2}\left(\nu \right)$, as well as $F_{2}^{>}(\nu )$, are monotonically decreasing. Thus, we obtain only one value for the NSeDD based on the solution stated in Equation (50), which always corresponds to the upper limit of the laser hazard zone.

#### 3.2.2. NSeDD Estimate for Laser Beams of Small Diameter

In the situation where the dazzle level of interest occurs at smaller diameters of the laser beam, i.e., when $\nu <\sqrt{2}$, we could not find a generally applicable estimate for the NSeDD, since the situation can become quite complex, as explained before. However, if a strong laser dazzle is assumed, i.e., for large values of the dazzle level, ϵ, an analytical approximation for the upper value of the NSeDD can be found. For this, we define two constraints:

**Constraint 1:** We assume that the dazzle level, ϵ, is high or, more precisely, $\Theta_{\epsilon }\gg l$.

Thus, ${\left(\frac{\Theta_{\epsilon }}{\nu^{*}l}\right)}^{2}\gg 1$ and $K_{2}$ can be approximated by using the following:

(51) $$K_{2}^{<}\left(\nu \right)\approx \frac{2N_{ss}b_{0}}{\nu^{2}}{\left(\frac{\sqrt{2}\Theta_{\epsilon }}{\nu l}\right)}^{s}=2N_{ss}b_{0}{\left(\frac{\sqrt{2}\Theta_{\epsilon }}{l}\right)}^{s}\cdot \frac{1}{\nu^{2+s}\;}.$$

Using Equations (14) and (15), the constraint of $\Theta_{\epsilon }\gg l$ translates to

(52) $$\Theta_{\epsilon }\gg l\;\;⟺\;\;\epsilon \gg \frac{2fl}{N_{max}p}=\frac{l}{FOV/2}.$$

For example, using the parameters given in Table 2, the dazzle level, ϵ, should fulfill the following inequation:

(53) $$\epsilon \gg \frac{2fl}{\max\left(N_{col},N_{row}\right)\cdot p}=\frac{2\cdot 25\;mm\cdot 2.04\;mrad}{808\cdot 4.8\;µm}=0.026=2.6\%$$

**Constraint 2**: We assume that the term $K_{2}\left(\nu^{*}\right)\cdot f_{2}\left(\nu \right)$ dominates in the left-hand side of Equation (44),

(54) $$K_{2}\left(\nu^{*}\right)\cdot f_{2}\left(\nu \right){\gg K}_{1}\cdot f_{1}\left(\nu \right),$$

which means that we can neglect the term $K_{1}\cdot f_{1}\left(\nu \right)$ in Equation (44).

Since the maximum of $f_{1}\left(\nu \right)$ occurs for $\nu =\sqrt{2}$, we can rewrite constraint 2 given in Equation (54) as follows:

(55) $$K_{2}^{<}\left(\sqrt{2}\right)\cdot f_{2}\left(\sqrt{2}\right){\gg K}_{1}\cdot f_{1}\left(\sqrt{2}\right)$$

By using constraint 1, we obtain

(56) $$N_{ss}b_{0}{\left(\frac{\Theta_{\epsilon }}{l}\right)}^{s}\cdot \left(1-\frac{1}{e}\right)\gg \frac{\lambda F}{\pi^{3}f\Theta_{\epsilon }^{3}}\cdot \frac{1}{e}\;⟺\;\;\;\Theta_{\epsilon }^{s+3}\gg \frac{\lambda Fl^{s}}{\pi^{3}fN_{ss}b_{0}}\cdot \frac{1}{e-1}$$

and by neglecting 1/(e−1) ≈0.58 and using Equations (14) and (15), we finally come to

(57) $$\epsilon \gg \sqrt[{s+3}]{\frac{\lambda Fl^{s}}{\pi^{3}fN_{ss}b_{0}}}\cdot \frac{2f}{N_{max}p}$$

As an example, using the parameters given in Table 2, constraint 2 would demand that the dazzle level, ϵ, has also to fulfill the following inequation:

(58) $$\epsilon \gg \sqrt[{-1.86+3}]{\frac{532\;nm\cdot 1.8\cdot {\left(2.04\;mrad\right)}^{-1.86}}{\pi^{3}\cdot 25\;mm\cdot 14\cdot 6.92}}\cdot \frac{2\cdot 25\;mm}{808\cdot 4.8\;µm}=0.037=3.7\%$$

In this example, both constraints result in comparable minimum values of dazzle level, ϵ. Now, using these two constraints, we can go back to obtain an analytical estimate for the solution of Equation (44) for the case of $\nu <\sqrt{2}$:

Looking at Figure 9, we can see that we can roughly approximate $f_{2}(\nu )$ for small values of truncation factor $\nu <\sqrt{2}$ by using the following calculation:

(59) $$f_{2}\left(\nu \right)\approx F_{2}^{<}\left(\nu \right)=1.$$

$F_{2}^{<}\left(\nu \right)$ is also plotted in the graph of Figure 11. Using this approximation leads, in conjunction with constraint 1 and constraint 2, to the following simplified version of Equation (44):

(60) $$K_{2}^{<}\left(\nu_{hd}\right)=K_{3}\;⟺\;\;\;2N_{ss}b_{0}{\left(\frac{\sqrt{2}\Theta_{\epsilon }}{l}\right)}^{s}\cdot \frac{1}{\nu_{hd}^{2+s}\;}=\frac{E_{sat}f^{2}}{P_{0}T}$$

Finally, we obtain the following result:

(61) $$\nu_{hd}^{2}=2{ [\frac{N_{ss}b_{0}P_{0}T}{E_{sat}f^{2}}{\left(\frac{\Theta_{\epsilon }}{l}\right)}^{s}]}^{\frac{2}{2+s}}\;$$

It is important to note that we have to take care when we make use of the estimate delivered by Equation (61). Looking at Figure 10, we can see how to interpret this estimate:

- In the case of s>−2 (e.g., see Figure 10a), the course of $K_{1}\cdot f_{1}\left(\nu \right)+K_{2}\left(\nu^{*}\right)\cdot f_{2}\left(\nu \right)$ is monotonically decreasing. If Equation (61) delivers a result for $\nu <\sqrt{2}$, we should not obtain a result from Equation (50) for $\nu \geq \;\sqrt{2}$. Thus, the estimate of Equation (61) corresponds to the upper limit of the NSeDD.
- In the case of s<−2 (e.g., see Figure 10b), Equation (7) may deliver a solution. However, the course of $K_{1}\cdot f_{1}\left(\nu \right)+K_{2}\left(\nu^{*}\right)\cdot f_{2}\left(\nu \right)$ is increasing for $\nu <\sqrt{2}$, which means that we also expect a solution from Equation (50). In this case, the solution of Equation (50) corresponds to the upper limit of the NSeDD, whereas the solution of Equation (61) corresponds to the lower limit.

### 3.3. Remark Regarding the Application of the Approximative Equations

In order to obtain an estimate for the NSeHD and the NSeDD, the approximations of Equations (41), (50) and (61) can be used to calculate corresponding values, $\nu_{hd}$, of the truncation factor. The respective distances can be calculated from these values by using Equations (27) or (42).

However, the reader should be aware that the approximations may deliver values of truncation factor, ν, that are lower than the lowest possible truncation factor defined by the laser output diameter, $d_{0}$: $\nu_{min}=\frac{d_{0}}{d_{ap}}=\frac{d_{0}\cdot F}{f}$. In this case ($\nu_{hd}<\nu_{min})$, the result of the approximative equations is irrelevant and has to be discarded.

Furthermore, to avoid complex analyses in the case of laser dazzle, we recommend, for the sake of simplicity, to determine only an upper value of the NSeDD. For this purpose, Equations (50) and (61) should be evaluated to estimate possible values of $\nu_{hd}$, and then the maximum should be further used for calculating the NSeDD by using Equation (42).

Moreover, for the case of laser damage, we propose to estimate only the upper value for the laser hazard distance by using $\nu_{uhd}$ of Equation (41). Looking at Figure 5 and Figure 6, we can see that the lower value of the laser hazard distance is in the range of some meters. For practical laser safety outdoors, this may not be relevant.

## 4. Discussion

In Section 3, we derived closed-form approximations to estimate the NSeHD and the NSeDD. Although numerical calculations of these quantities may deliver more exact values, these approximations are quite useful when calculations need to be performed ad hoc in the field. It is important to note that the NSeHD/NSeDD calculations, whether numerically or analytically performed, are only as accurate as the underlying theoretical model.

In an earlier work, we compared the calculated spatial light distribution in the focal plane of a camera lens, based on our theoretical model, with the output of the optical design software FRED [25]. We showed that the results of our theoretical model are comparable with the simulated results. We therefore assume that our theoretical model is appropriate to perform laser safety calculations for imaging systems and, at this point, take a closer look at the accuracy of the analytical approximations derived in this publication.

In order to investigate the accuracy of our approximations regarding hazard or dazzle distances, we calculated the NSeHD and the NSeDD for a range of parameters, which are listed in Table 3. The calculations were performed both numerically and analytically. The analytical calculations were performed using the approximations of Equations (41), (50), and (61) for $\nu_{hd}$ and the subsequent calculation of the NSeHD and NSeDD by applying the calculated values of $\nu_{hd}$ to Equations (27) and (42), respectively. As can be seen in Table 3, we had a reduced parameter variation for the calculation of the NSeDD as compared to the NSeHD. This was necessary to reduce the higher computational effort in the case of the NSeDD, since a higher number of parameters had to be considered for this quantity.

From the results of the NSeHD/NSeDD calculation, we estimated the absolute and relative error of the approximation by using the following equations:

(62) $$\Delta NSeHD={NSeHD}_{appr}-{NSeHD}_{num}\;,\;\Delta NSeDD={NSeDD}_{appr}-{NSeDD}_{num}\;,\;\delta NSeHD=\frac{\Delta NSeHD}{{NSeHD}_{num}}\;,\;\delta NSeDD=\frac{\Delta NSeDD}{{NSeDD}_{num}}\;,$$

where the subscript ‘appr’ denotes the approximate hazard distance using the approximative equations, and the subscript ‘num’ denotes the numerically calculated hazard distance. We rate the results of our approximations as good if the relative error does not exceed the arbitrarily chosen value of 50%. However, the sign of the relative error is also of importance when it comes to laser safety. A positive relative error means that the approximative hazard distance is larger than the numerically calculated one, which is good in terms of laser safety. A negative relative error, however, would imply an underestimation of the hazard distance, which would be bad in terms of laser safety. We, therefore, established the following rating scheme for the results of our approximations:

- δNSeHD,δNSeDD<−0.5: poor;
- δNSeHD,δNSeDD∈[−0.5,0]: satisfactory;
- $\delta NSeHD,\delta NSeDD\in [0,+0.5]$: good;
- δNSeHD,δNSeDD>+0.5: sufficient.

**Figure 12:**
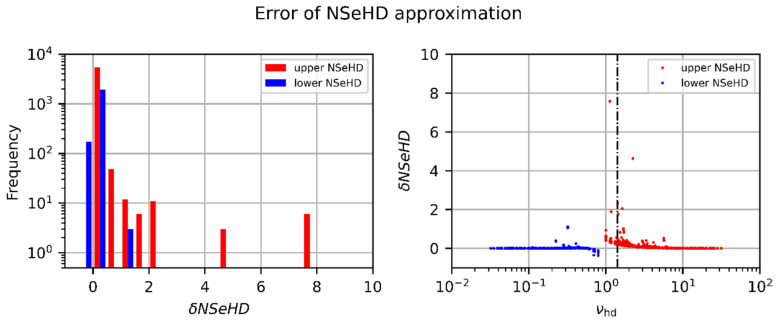
Relative error, δNSeHD, regarding the approximative NSeHD calculations according to Equation (62). The red and blue data points correspond to the upper and lower value of the hazard distance, respectively. Left: Histogram showing the frequency of the δNSeHD values. Right: δNSeHD as a function of the numerically estimated truncation factor, $\nu_{hd}$.

In Figure 12, the calculated values of the relative error, δNSeHD (regarding laser damage), are plotted in two different types of graphs. In both graphs, the red and blue color correspond to the upper and lower value of the hazard distance, respectively. The graph on the left-hand side shows a histogram of the values of δNSeHD (bin width 0.5); the graph on the right-hand side is a plot of the relative errors, δNSeHD, as a function of the numerically calculated value of the truncation factor, $\nu_{hd}$.

Looking at the histogram of Figure 12 and considering the logarithmic scale of the frequency axis, we can see that, for most calculated hazard distances, the relative error is near zero. Larger relative errors (up to a value of ~7.6) occur mainly for the upper value of the NSeHD. Far more interesting, however, is the sign of the relative error, which is clearly recognizable in the plot on the right-hand side. We can see that the relative error is mainly positive; negative relative errors occur only for lower values of the NSeHD. Furthermore, we can see that large relative errors mainly occur for small values of the truncation factor. The assignment of the calculated errors according to the rating scheme results in the classification shown in Table 4. From these results, we conclude that the approximative equations are well usable to determine hazard distances for sensor damage.

**Figure 13:**
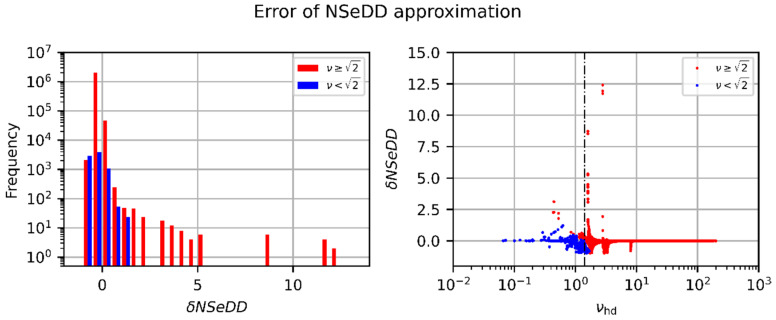
Relative error, δNSeDD, regarding the approximative NSeDD calculations according to Equation (62). The red and blue data points correspond to the approximations for large and low values of the truncation factor ($\nu \geq \sqrt{2}$ and $\nu <\sqrt{2}$), respectively. Left: Histogram showing the frequency of the δNSeDD values. Right: δNSeDD as a function of the numerically estimated truncation factor, $\nu_{hd}$.

In Figure 13, equivalent graphs are plotted for the NSeDD. In these graphs, the red color corresponds to the approximation for $\nu \geq \sqrt{2}$, calculated using Equation (50). The blue color corresponds to the approximation for $\nu <\sqrt{2}$, calculated using Equation (61). In the case of the NSeDD, we can see a similar distribution in the histogram, but not as good as for the NSeHD. Larger relative errors can occur for the approximation for $\nu \geq \sqrt{2}$. We can recognize in the graph on the right-hand side that these cases mainly occur for values of the truncation factor, $\nu_{hd}\approx \sqrt{2}$. Furthermore, we can see that, in the case of the approximative equations for calculating the NSeDD, the relative error is negative for a considerable amount of the calculations. For the NSeDD, the assignment of the calculated errors according to the rating scheme leads to the distribution shown in Table 5. In this case, we can conclude that the approximative equations have to be used with care for the estimation of hazard distances for sensor dazzle.

## 5. Summary

In this publication, we continued our work regarding laser safety calculations for electro-optical imaging systems. While our earlier publication [17] focused on the estimation of exposure limits for sensor damage and dazzle, here, we analyzed in detail the hazard distances resulting from these exposure limits. Following our earlier work, we tried to offer simple approximative closed-form equations to the system operator for the calculation of the hazard distances for electro-optical imaging systems. In addition, the accuracy of these approximations was examined. Our investigations predict interesting effects, like a lower limit of the laser hazard zone for electro-optical imaging systems, which is typically not existing in laser eye safety. The accuracy of our results, based on the approximative equations we deduced, was found to be very satisfactory in the case of sensor damage. Regarding sensor dazzle, there is still potential for improvement in the future. Future work may also include the experimental validation of the lower limit of the laser hazard zone for imaging systems, even though the lower hazard distance may only be of limited importance for practical laser safety.

Finally, we would like to summarize the most important steps and equations to perform a laser safety calculation for electro-optical imaging systems.

### 5.1. Summary of Equations Regarding Laser Damage

The exposure limit regarding laser damage ${MPE}_{S}$ can be estimated using Equation (12):

$${MPE}_{S}=E_{dam}\cdot \frac{16\lambda^{2}F^{4}}{T\pi^{2}f^{2}}{ [\frac{\frac{1}{\nu^{2}}}{1-\exp\left(-\frac{1}{\nu^{2}}\right)}]}^{2}$$

In order to find the corresponding laser hazard distance, NSeHD, for a specific laser source, first the truncation factor that corresponds to the upper value of the hazard distance has to be estimated using Equation (41):

$$\nu_{uhd}^{2}=\frac{P_{0}T\pi }{4E_{dam}\lambda^{2}F^{2}}+\sqrt{{\left(\frac{P_{0}T\pi }{4E_{dam}\lambda^{2}F^{2}}\right)}^{2}-1}$$

If the constraint $\nu_{uhd}>\nu_{min}=\frac{d_{0}\cdot F}{f}$ is fulfilled, the hazard distance can then be calculated using Equation (27):

$$NSeHD=\frac{\sqrt{\nu_{uhd}^{2}\cdot f^{2}/F^{2}-d_{0}^{2}}}{\Phi };$$

otherwise, NSeHD=0.

### 5.2. Summary of Equations Regarding Laser Dazzle

The exposure limit regarding the laser dazzle’s ${MDE}_{S}$ can be estimated for a specific dazzle level, ϵ, using Equation (17):

$${MDE}_{S}\left(\epsilon \right)=\frac{4E_{sat}F^{2}}{\pi T}\frac{1}{\frac{\lambda F}{\pi^{3}f\Theta_{\epsilon }^{3}}\cdot \exp\left(-\frac{2}{\nu^{2}}\right)+\frac{N_{ss}b_{0}}{{\left(v^{*}\right)}^{2}}{ [1+{\left(\frac{\Theta_{\epsilon }}{v^{*}l}\right)}^{2}]}^{\frac{s}{2}}\cdot \frac{\left(1-\exp\left(-\frac{2}{\nu^{2}}\right)\right)}{2/\nu^{2}}}$$

with

$$\Theta_{\epsilon }=\epsilon \cdot \frac{FOV}{2}\;\;\;and\;\;\;\nu^{*}=\min\left(1,\frac{\nu }{\sqrt{2}}\right).$$

In order to find the corresponding laser hazard distance, NSeDD, for a specific laser source, the truncation factor that corresponds to the upper value of the hazard distance has to be estimated by applying Equations (50) and (61):

$$\nu_{hd,1}^{2}=\frac{2P_{0}T}{E_{sat}f^{2}}\left(\frac{\lambda F}{\pi^{3}f\Theta_{\epsilon }^{3}}+N_{ss}b_{0}{ [1+{\left(\frac{\Theta_{\epsilon }}{l}\right)}^{2}]}^{\frac{s}{2}}\right)-2$$

$$\nu_{hd,2}^{2}=2{ [\frac{N_{ss}b_{0}P_{0}T}{E_{sat}f^{2}}{\left(\frac{\Theta_{\epsilon }}{l}\right)}^{s}]}^{\frac{2}{2+s}}$$

Then, by taking the maximum of both, we obtain the following:

$$\nu_{hd}^{2}=\max\left(\nu_{hd,1}^{2},\nu_{hd,2}^{2}\right)$$

If the constraint $\nu_{hd}>\nu_{min}=\frac{d_{0}\cdot F}{f}$ is fulfilled, the hazard distance can then be calculated using Equation (42):

$$NSeDD=\frac{\sqrt{\nu_{hd}^{2}f^{2}/F^{2}-d_{0}^{2}}}{\Phi };$$

otherwise, NSeHD=0.

Please note: The value for $\nu_{hd,2}^{2}$ is only valid if the constraint of Equation (53)

$$\epsilon \gg \frac{2fl}{\max\left(N_{col},N_{row}\right)\cdot p}$$

and the constraint of Equation (57)

$$\epsilon \gg \sqrt[{s+3}]{\frac{\lambda Fl^{s}}{\pi^{3}fN_{ss}b_{0}}}\cdot \frac{2f}{N_{max}p}$$

are fulfilled.

## Figures and Tables

**Table 1:** Parameters used for the laser safety calculations.

Symbol	Unit	Quantity
Laser		
$P_{0}$	W	Power
$d_{63}$	m	Beam diameter (1/e)
$d_{86}=\sqrt{2}d_{63}$	m	Beam diameter (1/e^2^)
$d_{0}$	m	Beam diameter at laser output
λ	m	Wavelength
Camera lens		
f	m	Focal length
$d_{ap}$	m	Aperture/entrance pupil diameter
$F=f/d_{ap}$		f-number
$N_{oe}$		Number of optical elements
$N_{ss}=2\cdot N_{oe}$		Number of scattering surfaces
T		Transmittance
s; $b_{0}$; l	-; sr^−1^; sr^−1^; rad	Scatter parameters
Image sensor/camera		
p	m	Pixel size
A	m^2^	Pixel area
C	e^-^	Saturation capacity
η		Total quantum efficiency
$t_{exp}$	s	Exposure time
$E_{sat}$	W/m²	Saturation threshold
$E_{dam}$	W/m²	Laser-induced damage threshold
$N_{col}$; $N_{row}$		No. of pixels per column and row
Miscellaneous		
Z	m	Distance
r	m	Radial coordinate
$\nu =d_{86}/d_{ap}$		Truncation factor
$P_{in}=P_{0}(1-exp(-2/\nu^{2}))\;$	W	Laser power entering the camera lens
$d_{spot}=k\lambda F$	m	Laser spot size in the focal plane
k		Spot size constant
μ	1/m	Atmospheric extinction coefficient

**Table 2:** Parameters used for the example calculations.

Laser	
Wavelength, λ	532 nm
$\mathrm{Output}\;\mathrm{power},\;P_{0}$	5 mW
$\mathrm{Output}\;\mathrm{diameter},\;d_{0}$	3.5 mm
Full angle divergence (1/e²), Φ	0.5 mrad
Lens	
Focal length, f	25 mm
f-number, F	1.8
$\mathrm{No}.\;\mathrm{of}\;\mathrm{optical}\;\mathrm{elements},\;N_{oe}$	7
Transmittance, T	0.89
Scatter parameter (@ 550 nm), s	−1.86
$\mathrm{Scatter}\;\mathrm{parameter}\;(@\;550\;\mathrm{nm}),\;b_{0}$	6.92 sr^−1^
Scatter parameter (@ 550 nm), l	2.04 mrad
Image sensor/camera	
$\mathrm{Size}\;(N_{col}$$\;\times \;N_{row}$)	808 px. × 608 px.
Pixel size, p	4.8 µm
Quantum efficiency, η	0.53
$\mathrm{Exposure}\;\mathrm{time},\;t_{exp}$	20 ms
Saturation capacity, C	7230
$\mathrm{Damage}\;\mathrm{threshold},\;E_{dam}$	73 kW/cm²

**Table 3:** Input parameters used to calculate numerically, as well as analytically, the NSeHD and NSeDD values.

Parameter	Parameter Variation for	
	NSeHD	NSeDD
**Laser**		
Wavelength, λ (nm)	400, 550, 700	
Divergence, Φ (mrad)	0.5, 1.0, 3.0	
$\mathrm{Laser}\;\mathrm{power},\;P_{0}$ (mW)	0.1, 5, 100	0.1, 5
Laser beam diameter, $d_{0}$ (mm)	1, 5, 10	1, 5
**Camera lens**		
Focal length, f (mm)	10, 25, 50, 100	10, 25, 100
f-number, F	1.4, 2.8, 5.6, 8, 16	1.5, 5.6, 16
Transmittance, T	0.6, 0.8, 1.0	0.6, 1.0
$\mathrm{No}.\;\mathrm{of}\;\mathrm{scattering}\;\mathrm{surfaces},\;N_{ss}$		10, 14, 20
Scatter parameter, s		−2.5, −2.0, −1.5
Scatter parameter, b (sr^−1^); see Equation (66)		0.1, 1.0, 3.0
Scatter parameter, l (mrad)		1, 2, 4
**Image sensor/camera**		
Pixel size, p (µm)	2, 5	
Quantum efficiency, η	0.6, 0.8	
$\mathrm{Exposure}\;\mathrm{time},\;t_{exp}$ (µs)	100, 5000	
$\mathrm{No}.\;\mathrm{of}\;\mathrm{pixels}\;(\mathrm{column}/\mathrm{row}),\;N_{pix}$	1000, 2000	
Laser-induced damage threshold, $E_{dam}$ (kW/cm²)	50, 75, 100	
Dazzle level, ϵ		0.2, 0.5, 1.0

**Table 4:** Rating of the results for the approximative equation to estimate the NSeHD. Please note: The results are based on a limited dataset of NSeHD calculations, as given by Table 3.

	Poor	Satisfactory	Good	Sufficient
	δNSeHD<−0.5	$\delta NSeHD\in [-0.5,\;0]$	$\delta NSeHD\in [0,\;+0.5]$	δNSeHD>+0.5
Upper NSeHD	-	-	98.41%	1.59%
Lower NSeHD	-	8.06%	91.80%	0.14%

**Table 5:** Rating of the results for the approximative equations to estimate the NSeDD. Please note: The results are based on a limited dataset of NSeDD calculations, as given by Table 3.

	Poor	Satisfactory	Good	Sufficient
	δNSeDD<−0.5	$\delta NSeDD\in [-0.5,\;0]$	$\delta NSeDD\in [0,\;+0.5]$	δNSeDD>+0.5
$\mathrm{Approx}.\;\nu \geq \sqrt{2}$	0.10%	97.70%	2.18%	0.02%
$\mathrm{Approx}.\;\nu <\sqrt{2}$	35.80%	49.86%	13.36%	0.98%

## Data Availability

The data presented in this study are available on request from the corresponding author.

## Appendix A. Non-Standard Parameters for Laser Safety Calculations

### Appendix A.1. Damage Thresholds of Image Sensors

Information on laser-induced damage thresholds of image sensors for continuous-wave (cw) laser radiation in the visible spectral range is rare. Publications related to measured cw-laser-induced damage thresholds (LIDTs) of CCD and CMOS cameras are, for example, Becker et al. [26,27], Théberge et al. [28], Burgess et al. [29], Westgate et al. [30], and Schwarz et al. [31,32,33] Here, we refer to the last-mentioned publication by Schwarz et al. and summarize these threshold values in Table A1. Please note that Schwarz measured these values for specific image sensors (CCD sensor: Sony ICX098, CMOS sensor: Aptina MT9V024). Laser damage thresholds for other types of image sensors may vary, but the order of magnitude (10–100 kW/cm²) should be similar.

**Table A1:** One-on-one laser-induced damage thresholds (LIDTs) measured for specific image sensors (CCD: Sony ICX098, CMOS: Aptina MT9V024) [31].

1-on-1 LIDT (kW/cm²)				
Image Sensor	Exposure Time (s)			
	0.25	1	5	10
CMOS, monochrome	75 ± 7	73 ± 15	56 ± 4	48 ± 3
CMOS, color	56.7 ± 1.8			
CCD, monochrome	146 ± 9	118 ± 9	93 ± 19	95 ± 21
CCD, color	14 ± 2	13 ± 2	11 ± 1	8.1 ± 0.8

### Appendix A.2. Saturation Thresholds of Image Sensors

The saturation threshold of an image sensor may be calculated using its technical specifications. At a specific irradiance, $E_{sat,pixel}$ (i.e., the saturation irradiance), the number of photoelectrons ($\mu_{e}$) generated by the incident photons within the camera’s exposure time ($t_{exp}$) will equal the saturation capacity (C) of a pixel. Using this relation, the saturation irradiance of a pixel of size p can then be estimated by the following equation:

(A1) $$E_{sat,pixel}=\frac{C\cdot hc/\lambda }{\eta \;A\;t_{\exp}},$$

where $A=p^{2}$ is the pixel area, η is the quantum efficiency, h is the Planck constant, and c is the vacuum speed of light.

Putting just the pixel saturation irradiance $E_{sat,pixel}$ from Equation (A1) into the ${MDE}_{S}$ equations (Equations (17)–(20)) would imply that the image sensor is illuminated by the dazzle laser only. In a real situation, the imaging system naturally observes a scene, which means that the charge generated by a pixel is determined by the ambient light of the scene and by the laser light. To meet this condition, we assume that the operator or the camera’s automatic exposure (AE) control will set the exposure time to a level, such that the image sensor’s mean pixel signal equals roughly half of the maximum pixel signal. Thus, by applying a factor of 0.5 to Equation (A1), we obtain an estimate for the saturation irradiance, $E_{sat}$:

(A2) $$E_{sat}\approx 0.5\cdot E_{sat,pixel}=0.5\cdot \frac{C\cdot hc/\lambda }{\eta \;A\;t_{\exp}}.$$

### Appendix A.3. Scatter Parameters

In our simple theoretical model of Section 2, the scatter component ($E_{s}$) contributing to the focal plane irradiance distribution ($E_{fp}$) depends on the scatter parameters (s, $b_{0}$, and l) of the lens used. Unfortunately, these scatter parameters are usually not known for COTS camera lenses. Thus, we performed a series of measurements where we recorded images of the focal plane irradiance distribution for a selection of eight typical camera lenses with focal lengths ranging from 25 mm to 100 mm [18]. We then derived radial irradiance profiles from the image data and subsequently fitted our model equations to these irradiance profiles, where we used the quantities s, $b_{0}$, and l as fit parameters. The outcome of this work was an individual set of scatter parameters for each camera lens that was under testing. We noticed that the scatter parameters for the different camera lenses are of the same order of magnitude and therefore additionally derived a generic set of scatter parameters based on statistical analyses of the individual sets of scatter parameters. This generic set may be applied together with our theoretical model to predict sensor incapacitation by laser light when a camera lens with unknown scatter parameters is used. For details, we refer the reader to Reference [18].

For this generic set of scatter parameters, we use capital letters:

(A3) $$S=-1.86;\;B=0.36\;sr^{-1};\;B_{0}=6.92\;sr^{-1};\;L=2.04\;mrad$$

The scatter parameters were validated by simulations with the FRED optical design software [25].

In Equation (A3), a fourth scatter parameter, B, is listed. This parameter refers to the scatter parameter b that is used in the two-parameter Harvey scatter model (see, e.g., Reference [20]) and can be used as an alternative for scatter parameter $b_{0}$. Both parameters are linked by the following equation:

(A4) $$b_{0}=b\cdot {\left(100l\right)}^{s}$$
